# The Chaotic Behavior of the Spread of Infection During the COVID-19 Pandemic in the United States and Globally

**DOI:** 10.1109/ACCESS.2021.3085240

**Published:** 2021-06-02

**Authors:** Nabin Sapkota, Waldemar Karwowski, Mohammad Reza Davahli, Awad Al-Juaid, Redha Taiar, Atsuo Murata, Grzegorz Wróbel, Tadeusz Marek

**Affiliations:** Department of Engineering TechnologyNorthwestern State University of Louisiana 5722 Natchitoches LA 71459 USA; Department of Industrial Engineering and Management SystemsUniversity of Central Florida 6243 Orlando FL 32816 USA; Industrial Engineering DepartmentTaif University 125895 Taif 26571 Saudi Arabia; MATériaux et Ingénierie Mécanique (MATIM)Université de Reims Champagne-Ardenne 27078 51100 Reims France; Department of Intelligent Mechanical SystemsGraduate School of Natural Science and TechnologyOkayama University 12997 Okayama 700-8530 Japan; Department of Logistics and Process EngineeringUniversity of Information Technology and Management in Rzeszów 49980 35-225 Rzeszów Poland; Department of Cognitive Neuroscience and NeuroergonomicsInstitute of Applied Psychology, Jagiellonian University 37799 31-007 Kraków Poland

**Keywords:** Chaotic behavior, COVID-19 pandemic, spread of infections, 0–1 test

## Abstract

In December 2019, China announced the breakout of a new virus identified as coronavirus SARS-CoV-2 (COVID-19), which soon grew exponentially and resulted in a global pandemic. Despite strict actions to mitigate the spread of the virus in various countries, COVID-19 resulted in a significant loss of human life in 2020 and early 2021. To better understand the dynamics of the spread of COVID-19, evidence of its chaotic behavior in the US and globally was evaluated. A 0–1 test was used to analyze the time-series data of confirmed daily COVID-19 cases from 1/22/2020 to 12/13/2020. The results show that the behavior of the COVID-19 pandemic was chaotic in 55% of the investigated countries. Although the time-series data for the entire US was not chaotic, 39% of individual states displayed chaotic infection spread behavior based on the reported daily cases. Overall, there is evidence of chaotic behavior of the spread of COVID-19 infection worldwide, which adds to the difficulty in controlling and preventing the current pandemic.

## I. Introduction

In December 2019, the coronavirus SARS-CoV-2 (COVID-19) broke out in China and soon grew into a global pandemic affecting almost every country worldwide [1]. The virus is transmitted through human-to-human contact, and infected individuals present with symptoms such as cough, fever, headache, and breathing difficulties [2]. Several elements facilitate the spread of infection and severity of disease: (1) close contact with infected individuals, (2) contact with individuals who have been to locations with a considerable number of confirmed COVID-19 cases, (3) efficiency and speed of COVID-19 transmission, (4) susceptibility of individuals over 65, and (5) having underlying health conditions (e.g., respiratory problems, hypertension, cardiovascular disease, and diabetes) [2].

Governments around the world have imposed different polices and undertaken preventive public health measures, such as social distancing orders, travel restrictions, local or national lockdown, and partial or complete border closures, to control the spread of the pandemic [3]. However, the COVID-19 pandemic remains one of the main sources of death worldwide at the time of writing this article. A better understanding of the underlying processes affecting COVID-19 pandemic dynamics and related infection patterns will help to improve the effectiveness of public health interventions worldwide.

Even though many different aspects of the COVID-19 pandemic have already been investigated, including the mechanisms and rate of infection transmission, how fast it reaches its peak, and how long it will last, it is difficult to accurately assess its dynamics [4]. Since the COVID-19 pandemic started in one or more specific places in China and quickly spread worldwide, it is reasonable to assume that it represents a nonlinear complex phenomenon. One of the main factors of complex systems is chaotic behavior, in which elements of the system align and compete for survival [5]. Sensitivity to initial conditions renders chaotic systems unpredictable, especially long-term; however, chaotic systems can be described by a few variables and equations [6]. Recently, Jones and Strigul [6] concluded that the chaotic behavior of the spread of COVID-19 represents a deterministic chaotic system. Such knowledge will help to better understand the unpredictable impact of small changes in a multitude of social, behavioral, economic, and political conditions on the dynamics of infection, which should be considered when creating public health management policies and mitigation strategies.

## II. Objectives

To investigate the chaotic behavior of the COVID- 19 pandemic, we analyzed data from a total of 214 counties and territories globally. Specifically, we aimed to confirm that the COVID-19 epidemic displays chaotic behavior based on the analysis of time-series data of reported daily infections. Firstly, we investigated the spread of infection in different US states, followed by its behavior in other countries around the world. The present article is structured as follows: the background section discusses the objectives and conclusions of published articles concerning the chaotic behavior of pandemics; the methodology section explains the binary 0–1 test for chaos using time-series data; the results section represents the outcomes of the 0–1 test; and the discussion section explains the results.

## III. Background

The dynamics of the spread of pandemics are similar to the behavior of other nonlinear systems including chaotic maps and turbulent flows [7]. In such systems, a small seed increases exponentially and then saturates; in general, chaotic behavior indicates that the system is extremely sensitive to the initial conditions. Some of the distinguishing features of nonlinear behavior can be used to analyze human pandemics. Many studies have investigated the deterministic chaotic dynamics of pandemics. Matouk [8] developed a susceptible-infected model with multi-drug resistance (SIMDR) and its fractional-order counterpart to predict the spread of the COVID-19 infection. In this compartmental model, the dynamics of one class of susceptible population and three classes of infected populations were investigated. The fractional-order counterpart of the model was considered with a view to achieving a higher degree of freedom and accuracy of modeling. Numerical tools of Lyapunov exponents (LEs), Lyapunov spectrum, and bifurcation diagrams were used to study the complex dynamics of the system. LE can be used to assess chaotic behavior by calculating the divergence rate of trajectories in the phase space; positive LE may indicate chaotic behavior, while negative LE does not generally represent stability [9]. The study by Matouk [8] verified the existence of chaotic attractors for the integer-order model and its fractional-order, and indicated that the fractional-order model was more realistic than its integer-order SIMDR model in explaining real epidemics data.

Other studies have investigated the chaotic behavior of different epidemics such as Rift Valley fever, childhood diseases, and epizootic outbreaks. Pedro *et al.* [10] developed a susceptible-asymptomatic-infectious-recovered compartmental model to investigate the Rift Valley fever epidemic. The study used a variety of nonlinear analysis tools, including bifurcation diagrams, maxima return maps, Poincaré maps, and LEs to investigate the nonlinear dynamics of the system. Billings and Schwartz [11] developed a susceptible-exposed-infectious-recovered (SEIR) model to forecast outbreaks of childhood diseases by adding stochastic perturbations to model parameters and LEs to identify chaotic behavior. Sun *et al.* [12] proposed a susceptible-infectious-recovered-susceptible (SIRS) model, and demonstrated the model’s chaotic properties based on LE indices. Eilersen *et al.* [13] described an eco-epidemiological model with a two-prey one-predator ecosystem, where one carries a disease. To assess whether the dynamic model is chaotic, the study determined the LE. Following an epizootic outbreak, chaotic behavior occurred in the system at different ranges of model parameters. The study indicated that chaos mostly occurs when the disease spreads into more prey species. Interestingly, the study verified chaotic behavior in a relatively minimal eco-epidemiological system [13].

Several articles have investigated chaotic behavior under specific environmental and geographic conditions, including the presence of noise, presence of seasonality, impacts of locations (small and big cities), and impacts of compartmental models. He and Banerjee [14] analyzed a fractional-order susceptible-infectious-recovered (SIR) epidemic system under the conditions of external noise and parametric seasonality. Although an integer-order SIR system exhibited stable behavior, the fractional-order SIR epidemic model in the presence of noise and seasonality forces showed strong patterns of nonlinear dynamic changes. This study utilized numerical tools, such as MFuzzyEn and the largest positive LE, and reported rich dynamic behaviors based on system parameters, degree of noise and seasonality, and fractional derivative order. The study concluded that the infectious disease outbreak could be controlled by applying efficient health and medical measures [14]. Yi *et al.* [15] investigated the dynamic behavior of an SEIR compartmental model with seasonality in relation to the transmission rate. The study applied bifurcation diagrams, Poincaré maps, LEs, and Lyapunov diagrams to investigate the periodic, chaotic, and hyperchaotic behaviors of the system. Grenfell *et al.* [16] used a time-series-susceptible-infected-removed (TSIR) model to determine the dynamic features of measles epidemics in various cities.

The study indicated that the epidemic model could accurately determine the dynamic features of the long-term behavior of measles in big cities. Based on LEs, the results indicated that small cities are more predictable and stable than larger cities. Li *et al.* [17] applied a numerical simulation and qualitative analysis to investigate a discrete-time susceptible-infectious (SI) compartmental model using the Euler scheme to transform the continuous model into a discrete epidemic model. The complex dynamics of the system were investigated through transcritical bifurcation, Hopf bifurcation, and flip bifurcation. The results indicated that the discrete-time epidemic model has a richer dynamic behavior than the corresponding continuous-time model [17].

Several of the reviewed studies utilized an assessment of LE to draw conclusions regarding evidence of chaotic system behavior. In general, a positive LE value is an indicator of the potential for deterministic chaos in a system whose equations are known; however, for the examined time-series data used in the present study, an LE >0 may not be a sufficient indicator of chaotic behavior since the system equations are unknown [18]. It should also be noted that other studies applied fractal analysis to assess the spread of COVID-19 around the world [19]–[21].

A powerful computational alternative to confirm chaotic system properties is a 0–1 test. This test, first proposed by Gottwald and Melbourne [22], [23] and later improved by Gottwald and Melbourne [24], does not rely on the underlying equation of a system. Also, the 0–1 test is not computationally intensive and does not require phase space reconstruction of the investigated system. The test results close to 1 indicate the presence of chaos, while closer to 0 indicates lack of chaos.

He *et al.* [25] used an SEIR compartmental model and applied a particle swarm optimization (PSO) algorithm to determine parameters of the model representing the evolution of the COVID-19 pandemic in Hubei province, China. The study also used the 0–1 test to verify the presence of chaos in the system, indicating that the system could produce chaotic behavior in the presence of seasonality and stochastic infection. Recently, Ahmed and Matouk [26] developed an antimicrobial resistance (AMR) model containing both classes of susceptible and resistant features. The study investigated complex dynamics including the existence of homoclinic connections, flip bifurcations, multiple closed invariant curves, and coexistence of multiple attractors. The 0–1 test was successfully applied to verify the existence of chaotic behavior in the system.

## IV. Methodology

This section outlines the methodology for the determination of deterministic chaos using the 0–1 test and discusses relevant COVID-19 infection time-series data.

### A. Determination of Deterministic Chaos Using the 0–1 Test

The applied 0–1 test procedure using the notation outlined by Gottwald and Melbourne [24] is provided in Appendix.

### B. COVID-19 Data Source and Processing

The data used in the present study were collected from Johns Hopkins University (JHU) Center for Systems Science and Engineering (CSSE) COVID-19 Data [27], available in the GitHub online publication by Dong *et al.* [28]. The files used were ‘time_series_covid19_confirmed_US.csv’ (i.e., US data), containing county-level confirmed cases of COVID for all US states and territories, and ‘time series_covid19_deaths_global. csv’ (i.e., global data), containing similar data for countries and territories worldwide.

The aggregated data sources for these data included World Health Organization (WHO), European Centre for Disease Prevention and Control (ECDPC), US Center for Disease Control (CDC), BNO News, WorldoMeters, COVID Tracking Project, Los Angeles Times, and The Mercury News. In addition, the data provided by the entities authorized to release COVID-19-related data in each country worldwide were utilized. For US states and territories, data released by the county-level authorized bodies were used as the source of the COVID-19 data. These data were adjusted accordingly if any discrepancy was observed or reported. As of 4/26/2021, Google Scholar revealed a total of 4,170 cited data sources [28] when a search using the phrase “An interactive web-based dashboard to track COVID-19 in real-time” was used. Hence, we were able to assure the desired level of data quality control and the upkeep of the data used in the present study.

Data processing was performed using MATLAB (ver. R2020) and pandas (ver. 1.2.0), an open-source data analysis and manipulation library in Python programming language. The two databases used in this study provide the cumulative confirmed cases of COVID-19 by date. For example, daily cumulative COVID-19 time series data can be mathematically shown as:

\begin{equation*} y_{t}=\sum \nolimits _{i=1}^{t} y_{i}^{d} \quad \forall t\in T\tag{1}\end{equation*}

where $y_{t}$ is the cumulative count of COVID-19 confirmed cases for day $t$, $y_{t}^{d}$ is the daily count of confirmed COVID-19 cases at any day $t$, and $T$ is the number of days contained in the duration of the time series data, i.e., $t =1, 2,3,\ldots,T-2$, $T-1$, $T$. To calculate $y_{t}^{d}$, one can simply use the following mathematical operation.

\begin{equation*} y_{t}^{d}=y_{t}-y_{t-1}\quad \forall t\in T\tag{2}\end{equation*}

In this study, the US data between 1/22/2020 and 12/13/2020 (a day before the COVID vaccination started in the US) were selected for analysis. These county-level cumulative data were first converted into the daily count of confirmed cases using (2) for each county and later grouped by state and territory to secure one time-series data per state/territory for the date range mentioned above. A similar approach was utilized for the global data to identify the daily count of confirmed COVID-19 cases.

## V. Results

The results of the 0–1 test for chaotic behavior of the spread of COVID-19 infection based on the daily count of confirmed cases in the US and globally are discussed below.

### A. Spread of COVID-19 Infection in the US

For the US, the time-series data for each state from the day of the first confirmed case (COVID-19 infection) were used. The K-median values from the 0–1 test were used to signify whether the time-series infection data exhibited deterministic chaos. The time-series data with K-median values at or greater than 0.9 were classified as chaotic, and those with K-median values less than 0.9 were classified as non-chaotic.

**FIGURE 1.**
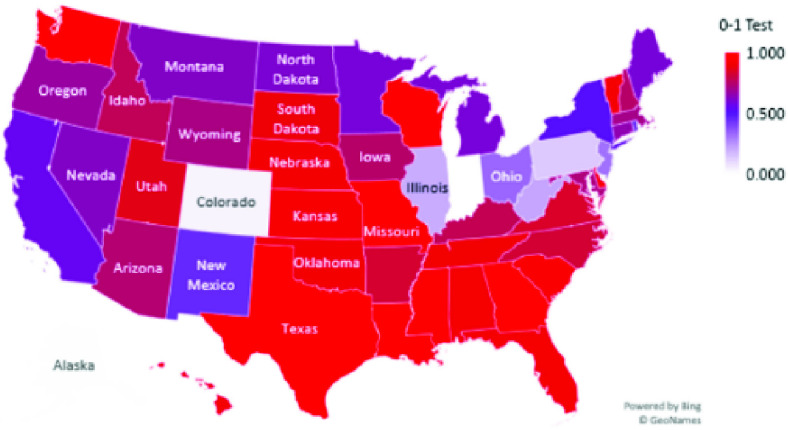
K-Median Values From the 0–1 Test for Confirmed Daily COVID-19 Cases in the US.

The results show that the spread of COVID-19 infection in 19 out of 50 states was chaotic (39.2%). The states that showed chaotic behavior in the examined time-series data for daily infections were Alabama, Delaware, District of Columbia, Florida, Georgia, Hawaii, Kansas, Louisiana, Mississippi, Missouri, Nebraska, Oklahoma, South Carolina, South Dakota, Tennessee, Texas, Vermont, Washington, and Wisconsin. Table 1 shows the K-median values from the 0–1 test for the confirmed daily COVID-19 cases by US state. In addition, Figure 1 illustrates a geographical map of US states, with the red color signifying chaotic behavior of the time-series data representing daily spread of infections.

**TABLE 1:** K-Median Values From the 0–1 Test for Confirmed Daily COVID-19 Cases in the US by State

States	K-median	Population Estimate	Total Confirmed Cases
Georgia	1	10,617,423	54,284
DC.	0.9761	705,749	2,487
Texas	0.9721	28,995,881	140,911
Wisconsin	0.9666	5,822,434	46,896
Louisiana	0.9664	4,648,794	26,861
Missouri	0.962	6,137,428	35,318
Washington	0.9583	7,614,893	20,206
Mississippi	0.9511	2,976,149	17,945
South Carolina	0.935	5,148,714	25,221
Delaware	0.9277	973,764	4,546
Kansas	0.921	2,913,314	18,846
Nebraska	0.9161	1,934,408	14,886
Alabama	0.9152	4,903,185	29,563
Vermont	0.9145	623,989	575
Florida	0.9133	21,477,737	112,593
Oklahoma	0.9088	3,956,971	23,767
Hawaii	0.9086	1,415,872	1,959
Tennessee	0.9051	6,829,174	45,431
South Dakota	0.901	884,659	9,104
Utah	0.86	3,205,958	23,390
Virginia	0.8332	8,535,519	28,191
Arkansas	0.8224	3,017,804	18,570
North Carolina	0.817	10,488,084	43,660
Kentucky	0.7762	4,467,673	22,310
Idaho	0.7711	1,787,065	12,118
Iowa	0.7473	3,155,070	25,625
New Hampshire	0.747	1,359,711	3,096
Arizona	0.7394	7,278,717	40,844
Maryland	0.728	6,045,680	23,465
Wyoming	0.7137	578,759	3,936
Massachusetts	0.7057	6,892,503	29,058
Oregon	0.6744	4,217,737	9,385
Connecticut	0.6512	3,565,287	14,676
Nevada	0.6224	3,080,156	18,683
Montana	0.6222	1,068,778	7,330
North Dakota	0.607	762,062	8,787
Minnesota	0.5992	5,639,632	37,882
Michigan	0.5982	9,986,857	46,516
Maine	0.5969	1,344,212	1,592
California	0.5585	39,512,223	158,538
New York	0.5145	19,453,561	77,516
New Mexico	0.4502	2,096,829	11,980
Rhode Island	0.4467	1,059,361	7,082
Ohio	0.3307	11,689,100	56,273
New Jersey	0.3113	8,882,190	40,065
West Virginia	0.212	1,792,147	6,322
Illinois	0.1971	12,671,821	84,890
Pennsylvania	0.1349	12,801,989	49,510
Colorado	0.0323	5,758,736	28,819
Alaska	0.0024	731,545	4,114
Indiana	0	6,732,219	42,543

**FIGURE 2.**
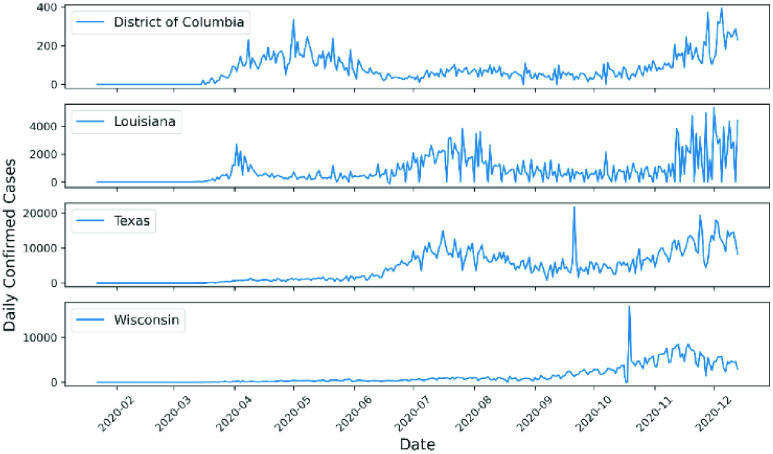
Examples of confirmed daily COVID-19 cases for states showing chaotic behavior.

**FIGURE 3.**
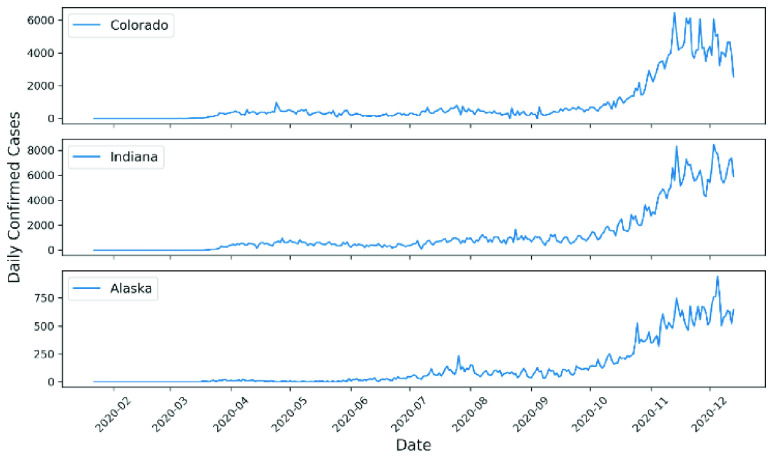
Examples of confirmed daily COVID-19 cases for states showing non-chaotic behavior.

### B. Spread of COVID-19 Infection on a Global Scale

**FIGURE 4.**
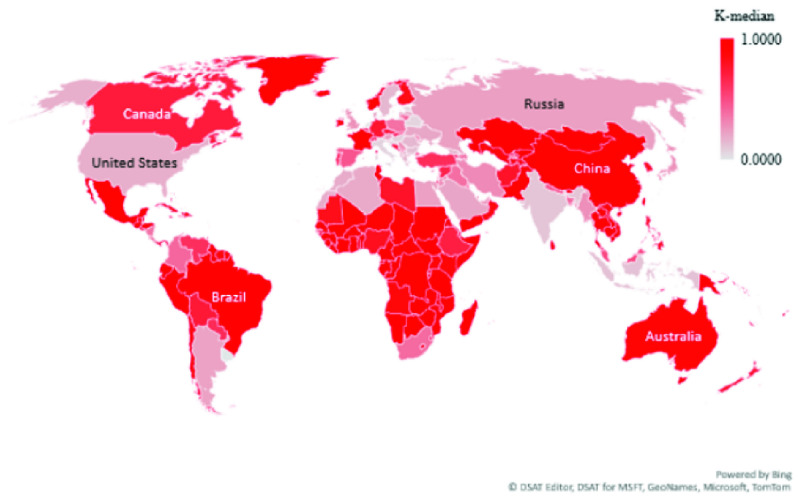
Results of the 0–1 test for confirmed daily COVID-19 cases globally.

**FIGURE 5.**
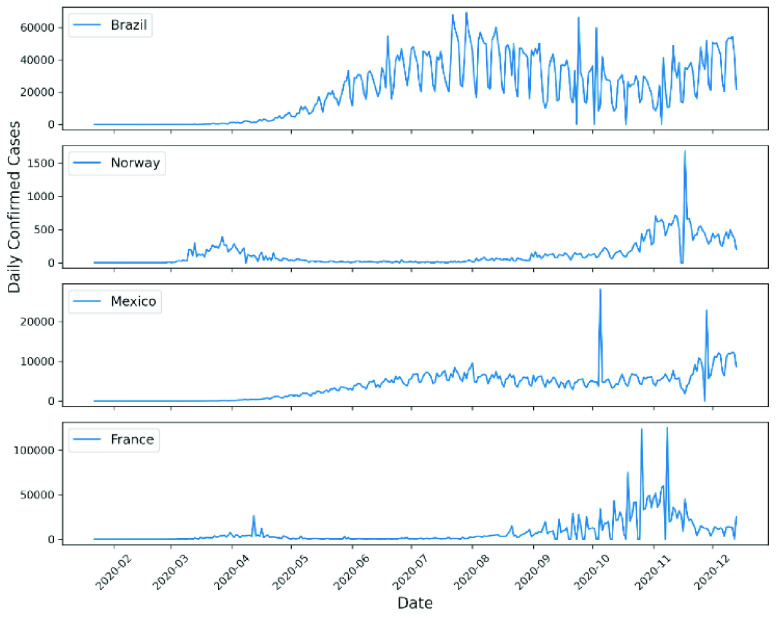
Examples of confirmed daily COVID-19 cases for countries showing chaotic behavior.

The results of the 0–1 test with K-median values for the daily count of confirmed cases of COVID in different continents, countries and territories on the global scale are depicted in Tables 2 and 3 and are illustrated in Figures 4–5. Figure 4 shows the geographical map with red color signifying chaotic behavior. Overall, 118 countries out of a total of 213 countries/territories (55%) exhibited chaotic behavior of the spread of COVID-19 infections. The Department of Economic and Social Affairs of the United Nations Secretariat (UN/DESA) classifies all countries and territories into three broad categories: 1) developed economies, 2) economies in transition, and 3) developing economies based on various econometric measures [29]. Table 4 shows that the proportion of developing countries or territories showing chaotic time series of daily confirmed COVID-19 cases was 68.3% (110 out of 161). In contrast, the same proportion for the developed and ‘in-transition countries or territories was 13.9% (5 out 36) and 18.8% (3 out of 16), respectively. This data indicates that Europe still was better off in terms of the proportion of countries with chaotic spread of infections (11 out of 32 or 34.4%). In contrast, the same figure was 86% for Sub-Sharan Africa and 70% for Latin America and the Caribbean (Table 5). The above provides convincing evidence that the likelihood of these countries and territories to have chaotic behavior in COVID-19 daily infection number is greater than that of their Asian counterpart, where this proportion was only 46.4% (13 out of 28), with Japan being designated as ‘developed’ country and removed from the calculation shown in Table 2.

**TABLE 2:** Results From 0–1 Test for Chaos for Daily COVID-19 Confirmed Cases Globally (Excluding the United States)

Continent/Subcontinent	Number of countries and territories	K-median >0.90	Percentage out of the group
Australia/New Zealand	2	1	50%
Central and Southern Asia	13	5	38%
Eastern and South-Eastern Asia	16	8	50%
Europe	48	14	29%
Latin America And the Caribbean	47	33	70%
Northern Africa And Western Asia	24	4	17%
Northern America	5	3	60%
Oceania (Excluding Australia And New Zealand)	8	7	88%
Sub-Saharan Afica	50	43	86%
Total:	213	118	55%

**TABLE 3:** Results of the 0–1 Test for Specific Countries/Territories (N = 213)

Country/Region	K-median
Solomon Islands	1
Antigua and Barbuda	0.999
Holy See	0.999
Kyrgyzstan	0.999
Saint Vincent and the Grenadines	0.999
Marshall Islands	0.999
Equatorial Guinea	0.999
Timor-Leste	0.999
Seychelles	0.998
Bhutan	0.998
Kazakhstan	0.998
Samoa	0.998
St. Martin	0.998
Barbados	0.997
Burundi	0.997
Sao Tome and Principe	0.997
Congo (Brazzaville)	0.997
Mayotte	0.997
Saint Barthelemy	0.997
Ecuador	0.997
Benin	0.997
Dominica	0.997
Guinea	0.996
Cambodia	0.996
South Sudan	0.996
Cameroon	0.996
Zimbabwe	0.996
Liberia	0.996
Comoros	0.995
Eritrea	0.995
Lesotho	0.995
Somalia	0.995
Mongolia	0.994
Saint Pierre and Miquelon	0.994
Cayman	0.994
Sudan	0.994
Sint Maarten	0.994
Peru	0.994
Togo	0.994
Tanzania	0.993
Burkina Faso	0.993
Sierra Leone	0.992
British Virgin Islands	0.992
Guadeloupe	0.992
Rwanda	0.992
Bahamas	0.992
Cuba	0.992
Fiji	0.991
Vietnam	0.991
Bonaire and Saba	0.991
Monaco	0.99
New Caledonia	0.99
Turks and Caicos Islands	0.99
Ghana	0.99
Gabon	0.99
Chad	0.99
Zambia	0.989
Gambia	0.989
Papua New Guinea	0.989
Laos	0.989
Congo (Kinshasa)	0.988
Faroe Island	0.988
Martinique	0.987
Guinea-Bissau	0.987
Trinidad and Tobago	0.987
Malawi	0.986
Gibraltar	0.986
Mexico	0.985
Yemen	0.984
Grenada	0.984
Central Afican Republic	0.984
Haiti	0.983
Pakistan	0.983
Norway	0.983
Guatemala	0.982
Djibouti	0.982
Botswana	0.982
Mali	0.982
Guyana	0.981
Jamaica	0.979
France	0.978
El Salvador	0.978
France	0.978
Taiwan	0.978
French Guiana	0.976
Cabo Verde	0.976
San Marino	0.975
Dominican Republic	0.973
Tunisia	0.972
Mauritius	0.972
Maldives	0.97
Brazil	0.969
Senegal	0.968
Bermuda	0.967
Honduras	0.967
French Polynesia	0.965
Madagascar	0.965
Greenland	0.965
Kosovo	0.963
Saint Kitts and Nevis	0.962
Eswatini	0.962
China	0.957
Saint Lucia	0.955
Andorra	0.954
Channel Islands	0.953
Namibia	0.949
Cote d’lvoire	0.949
Montserrat	0.949
Mozambique	0.94
Thailand	0.935
Iceland	0.927
Liechtenstein	0.925
Niger	0.919
Israel	0.917
Australia	0.914
Montenegro	0.907
Ireland	0.903
Panama	0.903
Uganda	0.901
Suriname	0.892
Malta	0.889
Belize	0.888
Kenya	0.888
Falkland Islands (Malvinas)	0.875
Diamond Princess	0.874
Uzbekistan	0.873
Angola	0.866
Sri Lanka	0.86
Tajikistan	0.859
Singapore	0.851
Oman	0.849
Chile	0.845
Philippines	0.839
Bahrain	0.835
Brunei	0.831
Finland	0.83
Kuwait	0.826
Curacao	0.824
Mauritania	0.815
Reunion	0.811
Luxembourg	0.8
Korea South	0.8
Nigeria	0.795
New Zealand	0.775
Libya	0.774
Afghanistan	0.772
Bosnia and Herzegovina	0.759
Germany	0.757
Czechia	0.747
Armenia	0.738
Bolivia	0.73
Slovakia	0.713
Canada	0.71
Ethiopia	0.693
Syria	0.668
Anguilla	0.644
MS Zaandam	0.639
Paraguay	0.636
Turkey	0.63
Venezuela	0.618
Portugal	0.615
Switzerland	0.606
Belgium	0.598
Malaysia	0.51
Cyprus	0.48
Spain	0.467
Nepal	0.457
Lebanon	0.436
Bangladesh	0.419
Lithuania	0.417
Moldova	0.415
Spain	0.407
Colombia	0.405
South Africa	0.392
Iraq	0.378
Poland	0.377
Qatar	0.367
Nicaragua	0.367
Costa Rica	0.353
Azerbaijan	0.351
Jordan	0.301
Iran	0.299
Serbia	0.292
United Arab Emirates	0.289
Burma	0.279
United Kingdom	0.277
Japan	0.269
Austria	0.245
Russia	0.231
Egypt	0.228
Argentina	0.223
Saudi Arabia	0.213
Ukraine	0.209
Algeria	0.193
Morocco	0.19
US	0.183
Denmark	0.177
Vanuatu	0.173
Latvia	0.171
North Macedonia	0.161
Georgia	0.157
Albania	0.157
Sweden	0.155
Romania	0.152
Greece	0.148
Slovenia	0.137
Bulgaria	0.125
Indonesia	0.121
India	0.114
Croatia	0.091
Hungary	0.083
Estonia	0.068
Italy	0.066
Belarus	0.065
West Bank and Gaza	0.057
Netherlands	0.017
Uruguay	0

**TABLE 4:** Economic Status of the Countries and Time Series Data of Daily-Confirmed Cases (Excluding the United States)

Economic Status	Number of countries or territories in the economic development category	K-Median > 0.9	
		Count	% within category
Developed countries	36	5	13.9%
Economy-in-Transition	16	3	18.8%
Developing countries	161	110	68.3%
Total	213	118	54.9%

## VI. Discussion

In the present study, only confirmed daily COVID-19 cases were subjected to nonlinear dynamics analysis. The available data for the number of hospitalizations, daily number of deaths, and number of recoveries were not considered here. While the time-series data for daily infections in the entire US were not found to be chaotic, results of the state-level analysis indicate that the confirmed COVID-19 infections for more than 35% of the states showed chaotic behavior. It should be noted that the US is one of the largest countries in the world and many of its states are comparable with other countries with respect to population size.

Given the fact that Italy, Spain, and the United Kingdom were greatly affected at the beginning of the pandemic in Europe, we also expected that such countries would also exhibit chaotic behavior in their reported daily infection data; however, this was not the case. It can be hypothesized that if the spread of infection in these countries was analyzed on a smaller scale— by recognized regions— the results may be different. For example, England (part of the UK) has nine recognized regions, and if London were to be considered separately, England alone would have ten-fold more data as compared with, for example, merged infection data for Northern Ireland and Scotland. In Asia, a similar situation could occur in countries such as India due to their large geographic size (28 states and 8 Union Territories) and massive population.

## VII. Limitations of the Study

We note several limitations of our methodology and results. The present study did not consider other important factors that may play a vital role in providing the ability to assess dynamics of the spread of COVID-19 infection, including for example, demographic and socioeconomic data such as income, education, employment, proportion of people under the poverty line, total population, population density, or urban vs. non-urban population. In addition, data regarding the relevant sociopolitical factors, government regulations, and public health policies should also be considered to improve our understanding of the nonlinear dynamics of the spread of COVID-19 infection worldwide and develop effective countermeasures. In future work, the largest Lyapunov exponent of each time-series data for daily infections should also be assessed to verify the current results.

## VIII. Conclusion

In the present study, time-series data representing the spread of infections based on the confirmed daily cases of COVID-19 during the period of 1/22/2020 to 12/13/2020 were investigated for the presence of chaos. The results showed that in most countries and territories irrespective of the continent, the data spread of COVID-19 infection exhibited chaotic behavior. The nature of deterministic chaos data renders it difficult to predict the dynamics of a pandemic in the long term [30].

Finally, our results confirm the conclusions made by Jones and Stirgul [6], who suggested that the COVID-19 epidemic demonstrates chaotic behavior that should inform future public health policies. Clearly, other data regarding the relevant sociopolitical factors, government regulations, and public health policies should be considered to improve our understanding of the nonlinear dynamics of the spread of COVID-19 infection worldwide and develop effective countermeasures. In addition, an evolutionary self-organizing map (ESOM) methodology [31] should be applied in the future to test the predictability of infection spread models.

## Appendix: Procedure for 0–1 Test

The 0–1 test procedure using the notation outlined by Gottwald and Melbourne [24] is as follows:

Step 1. Define a time series $x(t)$ for t = 1, $\ldots$, N, where $x(t)$ is the time-series value at time t. Additionally, define a set $C \in \boldsymbol \Re $ of non-negative random values. Gottwald and Melbourne [24] suggested random values for $C$ in the interval (0, $\pi$); therefore, in the present study, values for $C$ are in the interval ( $\pi $/5, $4\pi $/5) to avoid resonances distorting the statistics. While $c = 0$ must be avoided, and exclusion of $c=\pi $, is not necessary, the authors found the above procedure helpful.
Step 2. For each $c$: $c\in C$, and $n = 1, 2, \ldots, N$, compute the translation variables $p_{c}(n)$ and $q_{c}(n)$ as follows:
  \begin{align*} p_{c}(n)=&\sum \nolimits _{t=1}^{n} {x(t)\cos tc},\quad and q_{c}(n) \\=&\sum \nolimits _{t=1}^{n} {x(t)\,\mathrm {sin}\,tc}, for n = {\it 1,2,\ldots,} N\tag{3}\end{align*}
  After step 2, this procedure generates $N$ sets $p_{c}(n)$ and $q_{c}(n)$ for each $c$: $c\in C$ considered.
Step 3. Compute the mean square displacement for the translation variables (i.e., Fourier coefficients) $p_{c}(n)$ and $q_{c}(n)$ as follows:
  \begin{align*}&\hspace {-0.5pc}M_{c} (n) = \lim \limits _{N\to \infty }\frac {1}{N}\sum \nolimits _{t=1}^{N} [p_{c}\left ({t+n }\right)-p_{c}\left ({t }\right)]^{2} \\& \qquad \qquad\qquad\qquad\qquad\qquad +[q_{c}\left ({t+n }\right)-{\mathrm { }q}_{c}(t)]^{2}\tag{4}\end{align*}
  According to Gottwald and Melbourne [23], [24], for the time series with regular dynamics, the mean square displacement is a bounded function in time whereas it scales linearly with time ( $n$) if the time series is chaotic. In such a case, the plot of $p_{c}(n)$ vs. $q_{c}(n)$ looks like an irregular not bounded function in time. Gottwald and Melbourne [24] suggested that this definition of mean square displacement requires that be much less than $N$ or $n \ll N$. Gottwald and Melbourne [24] also suggested selection of $n$ such that $n < n_{\mathrm {cut}}$ where $n_{\mathrm {cut}} = N/10$, and reported that this scheme of selecting $n$ produced good results (plots) showing regular dynamics and chaotic dynamics clearly in a $p_{c}(n)$ vs. $q_{c}(n)$ graph.
Step 4. The $M_{c}(n)$ vs. $n$ plot shows increasing linearity with $n$; however, there is an oscillation confounded with $M_{c}(n)$. To smooth this oscillation, Gottwald and Melbourne [24] suggested using the modified mean square displacement for the translation variables, which can be defined as follows:
  \begin{equation*} D_{c} (n) = M_{c} (n)-V_{\mathrm {osc}} ({\it c,} n)\tag{5}\end{equation*}
  where $V_{\mathrm {osc}}$ ( $c$, $n) =({\textit{Ex}})^{2} \frac {1-\cos {nc}}{1-\cos c}$ and Et $=\lim \limits _{N\to \infty } {\frac {1}{N}} {\sum \nolimits _{t=1}^{N} {x(t)}}$
Step 5. Finally, $D_{c}(n)$ s values from step 4 were used to estimate the asymptotic growth rate, $K_{c}$. Using the correlation method, the asymptotic growth rate $K_{c}$ can be calculated as follows:
  \begin{equation*} K_{c} = \frac {\mathrm {cov}(\xi,\Delta)}{\sqrt {\mathrm {var}\left ({\xi }\right)\mathrm {var}(\Delta)}}\mathrm {\in [-1, 1]}\tag{6}\end{equation*}
  where $\xi = (1, 2, \ldots, n_{\mathrm {cut}})$ and $\Delta = (D_{c}$ (1), $D_{c}$ (2), $\ldots $, $D_{c}(n_{\mathrm {cut}}))$ and cov( $\xi $, $\Delta$) is the covariance between vectors $\xi $ and $\Delta $. Finally, Gottwald and Melbourne [32] also provided theoretical proof that their test yields a value of 0 for periodic and quasi-periodic dynamics and a value of 1 for nonlinear (chaotic) systems.
